# Instructing students to measure their own bone density and prepare a simulated health class during pharmacy school improves their awareness and understanding of osteoporosis prevention

**DOI:** 10.1186/s40780-016-0045-8

**Published:** 2016-05-03

**Authors:** Erisa Tomishige-Mukai, Akio Kawachi, Erika Kiyohara, Fuminori Esaki, Junichiro Sonoda, Tomohiro Shinya, Keiko Narumi, Keizo Sato, Toshiro Motoya

**Affiliations:** First Department of Clinical Pharmacy, School of Pharmaceutical Sciences, Kyushu University of Health and Welfare, 1714-1 Yoshino-machi, Nobeoka, Miyazaki 882-8508 Japan; Department of Clinical Biochemistry, School of Pharmaceutical Sciences, Kyushu University of Health and Welfare, Miyazaki, Japan

**Keywords:** Pharmacy education, Prevent osteoporosis, Measurement of bone density, Simulated-health class, Local residents

## Abstract

**Background:**

Osteoporosis is estimated to afflict over 200 million people worldwide and healthcare professionals are needed to successfully intervene. The aim of this study was to assess cognitive changes in students pertaining to the primary prevention of osteoporosis after measuring their bone density and having them participate in a simulated health class during pharmacy school.

**Methods:**

Third year pharmacy students participated in the training program, which consisted of measuring their bone density using quantitative ultrasound and preparing educational materials and conducting a simulated health class. The students’ knowledge concerning the prevention and education on osteoporosis was surveyed using questionnaires before and after the training.

**Results:**

The bone area ratio (BAR) in 24 % of the students was evaluated as category 4 (slightly low) or 5 (low or caution). Regression analysis indicated a significant relationship between the BAR and amount of exercise reported in both males (*p* = 0.005) and females (*p* = 0.004). The student-made educational materials were prepared in line with the requirements of the Japanese 2011 guidelines. The student response rates for the importance of food, exercise, and the bone density measurement in youth were significantly increased after the training (*p* < 0.001 in all). More than 95 % of students reported that the program was useful, improved their understanding, and important, with 94 % satisfied with the experience.

**Conclusions:**

This experience-based educational program combining measuring the bone density and the preparation and presentation of a simulated health class appeared to improve the awareness and understanding of osteoporosis prevention in pharmacy students.

## Background

Osteoporosis-related bone fractures can directly limit the activities of daily living (ADL) and reduce quality of life (QOL) [1], leaving certain patients bedridden. Osteoporosis is defined as “a skeletal disorder characterized by compromised bone strength predisposing a person to an increased risk of bone fractures” [2, 3], and it is estimated to afflict over 200 million people worldwide [4]. Therefore, osteoporosis prevention is extremely important to decrease the risk of osteoporosis-related bone fractures, and healthcare professionals are needed to successfully intervene.

Previous reports have shown that providing pharmaceutical care improves the health literacy of the patient [5, 6]. Accordingly, our group has delivered many presentations on osteoporosis prevention at local community centers to enhance the residents’ awareness of osteoporosis, in cooperation with the city office [7]. Yuksel et al. [8] reported that multifaceted interventions, including printed materials, a tailored educational program, and radiation-free bone density measurement using the quantitative ultrasound (QUS) method, at the community pharmacy leads people to obtain more precise bone mineral density testing, such as by the central dual energy X-ray absorptiometry (DXA) method, and the initiation of new prescriptions for osteoporosis. Therefore, activities that raise awareness of osteoporosis prevention combined with bone density measurements may further enhance the educational effects of community interventions.

We provided such a training program that combined the “QUS experience” and “a simulated health class for local residents” on the subject of osteoporosis prevention to students in the pharmacy school to improve their awareness and understanding of osteoporosis prevention. In the present study, we evaluated the cognitive changes in the students after training pertaining to the primary prevention of osteoporosis, as well as their satisfaction with and opinions on the training program. We also analyzed several different factors that influence bone status in youth.

## Methods

### Design of the training program

**Fig. 1:**
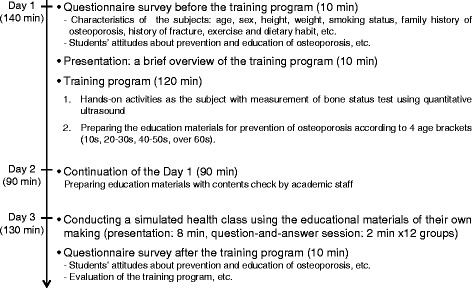
Schedule for the “osteoporosis prevention education for local residents” training program for pharmacy students

The training program “osteoporosis prevention education for local residents” was provided to 3rd year pharmacy school students at the School of Pharmaceutical Sciences, Kyushu University of Health & Welfare, during the comprehensive study classes offered in April 2013. The training program schedule is shown in Fig. 1. The training was undertaken on April 18th, May 9th and 16th (day 1: 140 min, day 2: 90 min, day 3: 130 min). The program consisted of measuring the bone density of the students and having them prepare educational materials on the prevention of osteoporosis for four age brackets (10s, 20–30s, 40–50s, over 60 years) and conduct a simulated health class using Microsoft PowerPoint slides of their own making. We divided the students into 12 groups of 8–9 people and assigned each age bracket to three groups. The academic staff provided students with guidance on the bone density measurement and on making the content of their educational materials. They also dealt with any questions on the spot.

### Measurements and statistical analysis

We assessed the characteristics of the students including sex, age, body-mass index (BMI), smoking status, history of bone fracture, family history of osteoporosis, amount of exercise, diet, and bone area ratio (BAR). The BAR at the calcaneus was evaluated using QUS (Benus α; Ishikawa Seisakusho Ltd., Hakusan, Japan) and was classified as category 1 to 5 (1: sufficient, 2: upper half of the average range, 3: lower half of the average range, 4: slightly low, and 5: low or caution) with the maker’s instruction manual, based on the study conducted by Kagechika et al. [9] and Yamamoto et al. [10]. Generally, the speed of sound (SOS) measured by QUS at the calcaneus shows a significant decline with age [11, 12], and Yamazaki showed that the elderly’s SOS was decreased by approximately 3.0 standard deviations compared from the young adult mean [13]. As the BAR calculated by SOS also goes through the same motion with SOS, the category 5 of BAR was set at −3.0 and −2.5 SD for male and female from the peak BAR of young adult, and the category 1–4 regions of the BAR were divided based on the mean value of BAR with age-dependent decline in a linear fashion and the range of SD respectively (male: 3.7 %/SD, female: 3.4 %/SD). In addition, we surveyed the students’ knowledge about the prevention and pathology of osteoporosis before and after the training. The program itself was also evaluated by the students using a subjective estimate of usability and satisfaction. For these measures, the student responses were scored using a five-point Likert scale (1: strongly disagree, 2: disagree, 3: neither, 4: agree, and 5: strongly agree). A 10-min self-administered survey containing the above-mentioned items was distributed to the 105 students who participated in the training, and the questionnaires were filled out anonymously. The responses in the 100 fully completed questionnaires were analyzed. Regression analysis was used to find significant associations between the BAR and the characteristics of the students. A Wilcoxon signed-rank test was used to assess differences between before and after the training. Associations between BAR (dependent variable) and amount of exercise (explanatory variables) were assessed using multiple regression analysis, and adjusted-R^2^ (coefficient of determination) and *β* (standardized partial regression coefficient) and *P*-values were estimated. *P*-values less than 0.05 were considered statistically significant. All statistical analyses were performed in IBM® SPSS® Statistics Version 21.0. The protocol for this study was approved by the ethics committee of Kyushu University of Health and Welfare (No.12–006), and the written informed consent was obtained from the pharmacy students.

## Results

The pharmacy student bone-related characteristics separated by sex for the 48 males and 52 females are shown in Table 1. The mean age and BMI were significantly greater in the males than in the females, and the incidence of smoking was also higher among males. Similar fractions of the males and females reported a history of fracture (39.6 % and 30.8 %) and family history of osteoporosis (4.2 % and 5.8 %). Significant differences in amount of exercise in the present and during high school were found between the sexes, but no significant differences were found during elementary and junior high school. Similar fractions of male (22.9 %) and female (32.7 %) students selected “well” or “slightly well” as their response to whether “you maintain a nutritious and balanced diet”.

**Table 1:** Pharmacy student bone-related characteristics by sex

Variables	Male (*n* = 48)		Female (*n* = 52)		*p* value^a^
Age, Mean (SD)	21.5	(3.0)	20.5	(1.7)	0.047
BMI, Mean (SD)	23.1	(4.3)	21.1	(4.2)	0.017
Smoking status, No. (%)					0.002
Never smoked	34	(70.8)	50	(96.2)	
Current smoker	11	(22.9)	2	(3.8)	
Former smoked	3	(6.3)	0	(0)	
History of fracture, No. (%)	19	(39.6)	16	(30.8)	0.356
Family history of osteoporosis, No. (%)	2	(4.2)	3	(5.8)	0.713
Amount of exercise (min/week) ^b^, Mean (SD)					
Present	130.5	(203.3)	39.5	(102.1)	0.005
During their high school days	550.4	(536.5)	313.3	(487.1)	0.023
During their elementary and junior high school days	796.5	(464.1)	608.0	(601.1)	0.084
Self-evaluation if they maintain nutritious and balanced diet (carbohydrate, protein and fat etc.), No. (%)					0.212
Well	4	(8.3)	7	(13.5)	
Slightly well	7	(14.6)	10	(19.2)	
Neither	15	(31.3)	14	(26.9)	
Not so good	15	(31.3)	19	(36.5)	
Poor	7	(14.6)	2	(3.8)	

^a^ *P-*values for comparisons between males and females using unpaired t-tests, Chi-square tests, or Mann–Whitney U-tests

^b^Amount of exercise accumulated throughout a week

**Fig. 2:**
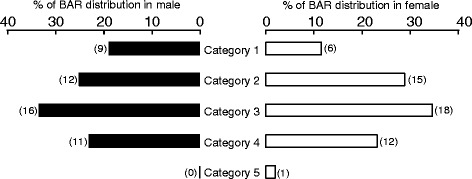
The distribution of BARs in males and females. The BARs were classified into five categories (Category 1: sufficient, 2: upper half of the average range, 3: lower half of the average range, 4: slightly low, and 5: low or caution)

The distributions of BAR in males and females are shown in Fig. 2. Importantly, 22.9 % of males and 25.0 % of females were classified as category 4 or 5, meaning “slightly low” or “low or caution”.

As shown in Table 2, the regression analysis showed a weak association between the BAR and amount of exercise (male: R^2^ = 0.121; female: R^2^ = 0.117), and indicated a significant relationship between the BAR and amount of exercise in the present for males (*β* = 0.419; *p* = 0.005) and females (*β* = 0.488; *p* = 0.004).

**Table 2:** The associations between BAR and amount of exercise (min/week)

	Dependent variable			
	BAR			
	Males (48)		Females (52)	
Adjusted R^2^	0.121		0.117	
Explanatory variables	β^a^	*p* value	β^a^	*p* value
Amount of exercise during their elementary and junior high school days	−0.002	0.987	−0.013	0.933
Amount of exercise during their high school days	−0.007	0.963	−0.168	0.357
Amount of exercise in the present	0.419	0.005	0.488	0.004

^a^ β: standardized partial regression coefficient

The content outline for the “education materials of osteoporosis prevention” made by the students for the four age brackets are shown in Table 3. On the slide-show presentation for youth in their 10s, the students included the following topics: the importance of regular food, physical activities, and adverse factors for bone growth with concrete examples provided. As the target subject age advanced, the students began addressing the following: the increased risk of osteoporosis in females compared with males, issues related to females such as pregnancy, lactation, and menopause, among others. The educational material for the over 60 group included information on the treatment of osteoporosis and the risk and prevention of osteoporosis-related bone fractures.

**Table 3:** The content outlines of simulated health class by the pharmacy students for each age bracket

The age bracket (group ^a^)	The content outline
10s (group 1–3)	• 10s is a crucial time for the bone growth and development. • A comparison of bone between young adults and osteoporosis patients. • Importance of regular food and physical activities for bone development. • Explanation of the nutrient required for bone development using some examples of food. • Adverse factors for the growing bones and the mechanism of bone loss induced by the following; smoking, alcohol, physical inactivity, excessive dieting, instant and snack food etc.
20s and 30s (group 4–6)	• 20s and 30s are crucial times for the maintenance of healthy bones. • Bone density is achieved a peak in 20s, and has declined progressively with age. • Women are at greater risk of developing osteoporosis than men. • Association between osteoporosis and female hormone estrogen. • Bone density during pregnancy and lactation. • Importance of prevention bone loss from earlier years. • Explanation of the nutrient required for the maintenance of healthy bones. • Risk factors of bone loss as follows; smoking, alcohol, physical inactivity and sun avoidance.
40s and 50s (group 7–9)	• Women need especially cautions by reason of severe bone loss after menopause. • Symptoms of osteoporosis; lower backache, stooped posture, fracture easily with progressive osteoporosis. • Early detection and rapid cure improve osteoporosis, and its screening test is necessary before people break a bone. • Food, physical activity and sun exposure promote bone health, and concrete numerical goals of the nutrient intake, physical activity and sun exposure were showed. • Protection of bone health is conductive to maintain general health.
Over 60s (group 10–12)	• The causes of osteoporosis and the method to halt the progression of bone loss with advancing age. • Symptoms suspected of osteoporosis; stooped posture and height loss etc. • Importance of osteoporosis screening test for early detection and rapid cure. • A provision to prevent a fall is critical tasks to avoid becoming bedridden by fracture. • Food, physical activity and sun exposure promote bone health, and concrete numerical goals of the nutrient intake, physical activity and sun exposure were showed. • Drug for the treatment of osteoporosis.

^a^Each group was composed of eight or nine pharmacy students, and each age bracket was assigned to three groups

The pharmacy student scores on food, exercise, and the bone density measurement in youth before and after the training are shown in Table 4. Before training, 92 % of students selected “strongly agree” or “agree” as their response to the statement “People need to take food with an eye on the nutrition” and 77 % similarly agreed with “People need to form an exercise habit” for the prevention of osteoporosis. After the training, those response rates were 100 % and 96 % (both *p* < 0.001). In addition, the percentage of students who selected “strongly agree” as their response to the statement “Countermeasures to prevent osteoporosis in 10s–20s are important” increased from 56 % to 92 % after training (*p* < 0.001).

**Table 4:** Pharmacy student responses question for prevention of osteoporosis before and after the training program

	Students responses before the training, %					Students responses after the training, %					
Question items ^a^	1	2	3	4	5	1	2	3	4	5	*p* value ^b^
People need to take food with an eye on the nutrition for the prevention of osteoporosis.	1	3	4	26	66	0	0	0	10	90	<0.001
People need to form an exercise habit for the prevention of osteoporosis.	1	4	18	27	50	0	1	3	13	83	<0.001
Countermeasures to prevent osteoporosis in 10-20s are important.	1	3	6	34	56	0	0	3	5	92	<0.001

^a^Each item was measured on a five-point Likert scale (1: strongly disagree, 2: disagree, 3: neither, 4: agree, and 5: strongly agree)

^b^ *P-*values indicate comparisons between the student responses before and after the training using Wilcoxon signed-rank tests

The pharmacy student responses evaluating the training program are shown in Table 5. For the statement “The training program was useful for you”, 96 % of the students answered “strongly agree” or “agree”, and 96 % of students agreed or strongly agreed that “The training program led to a better understanding of osteoporosis” and “Preventive education for osteoporosis like ‘a simulated health class’ on the training program was important for local residents”. In response to the statement “Learning about your own bone status data was useful for the prevention of osteoporosis”, 97 % of the students selected “strongly agree” or “agree”. Overall, 94 % of the students indicated satisfaction with the training program.

**Table 5:** Pharmacy student responses evaluating the training program

	Students responses, %				
Question items^a^	1	2	3	4	5
The training program was useful for you.	0	0	4	12	84
The training program led to a better understanding of osteoporosis (symptom, cause and prevention etc.).	0	1	3	9	87
Preventive education for osteoporosis like “a simulated health class” on the training program was important for local residents.	0	1	3	18	78
Learning about your own bone status data was useful for the prevention of osteoporosis.	0	0	3	7	90
You were satisfied with the training program on the whole.	1	0	5	25	69

^a^Each item was measured on a five-point Likert scale (1: strongly disagree, 2: disagree, 3: neither, 4: agree, and 5: strongly agree)

## Discussion

We provided a training program consisting of a simulated osteoporosis prevention class to students in pharmacy school and measured their bone density. Although the 20s is the most critical time for acquiring PBM, 24 % of the student BARs were evaluated as category 4 or 5 (slightly low or low or caution). Those students may have felt a sense of crisis because of their own measurement, resulting in changes in their awareness of the bone status in youth. According to the Dale’s Cone of Experience [14], students who “role-play a situation” and “simulate a real experience” are able to retain more information than learning through listening, reading, or observation. Although not of proven efficacy with or without hands-on activities as the subject with measurement of bone status test, the experience-based education program providing the experience of bone density measurement seemed to improve student understanding of osteoporosis by inducing “direct purposeful experience” regarding the prevention of osteoporosis.

The regression analysis indicated a significant relationship between the BAR and the amount of exercise in the present for both males and females. These results are consistent with those of a study by Miyabara et al. [15], which found that the present amount of exercise was strongly associated with the bone density of female college students. These data show that bone status is more closely related to the amount of present exercise than past. Because the amount of exercise generally decreases with increasing age in both males and females, maintaining a continuous exercise habit seems to be important for bone mass accumulation and osteoporosis prevention. From the current results of the extreme exercise loss after graduating high school and their bone status, the introduction of the physical education in the university curriculum as a part of health education may be worthy of consideration to prevent or alleviate oncoming of osteoporosis in aging process.

Students created their own educational materials on osteoporosis prevention after they measured their own bone density, and the outline of their content appeared to be in line with the requirements for each age bracket of the Japanese 2011 guidelines [3]. The educational materials designed by all groups included equivalent lifestyle choices, such as eating or exercise habits, and were slightly different for each age bracket.

According to the results of a survey conducted in Japan in 2009, the prevalence of osteoporosis increases with advancing age from middle-age to elderly in females and is higher in females than in males after age 60 [1]. The student-made educational materials were evaluated on the basis of including the background of osteoporosis for each age bracket. This suggested that the students understood the importance of educational interventions appropriate to each age bracket.

The intake of nutrients such as calcium and vitamins D and K is considered essential for the prevention and treatment of osteoporosis. All of the groups regardless of their assigned target age group included some form of food or nutrients in their slide-show presentation, and all students selected “strongly agree” or “agree” as their response to the statement “people need to take food with an eye on the nutrition for the prevention of osteoporosis” after the training, suggesting that the students deepened their understanding of the importance of healthy eating for the prevention of osteoporosis.

The percentage of students who responded “strongly agree” or “agree” to the statement “people need to form an exercise habit for the prevention of osteoporosis” was nearly 100 % after the training, up from about 80 % beforehand. Thus, most of the students with low amounts of exercise were likely awoken to the importance of physical activity.

For the statement “Countermeasures to prevent osteoporosis in 10–20s are important”, about half of the students selected “strongly agree” before the training, whereas almost 90 % did after the training. Before the training, the students may have falsely believed that osteoporosis is a disease of older people and may not have linked it to the PBM in a person’s youth. After training, most students seemed to understand the importance of acquiring as high a PBM as possible during one’s youth to avoid osteoporosis. Young people are rarely educated on osteoporosis or have the opportunity to measure their own bone density. Based on these results, we propose the necessity of an educational intervention for young people that include measuring their bone density.

In the present study, almost 100 % of the students responded “strongly agree” or “agree” to the utility of the training program and that it improved their understanding of osteoporosis. Moreover, for the statements “Preventive education for osteoporosis like ‘a simulated health class’ on the training program was important for local residents” and “Learning about your own bone status data was useful for the prevention of osteoporosis”, almost 100 % of students responded “strongly agree” or “agree”. Finally, 94 % of the students indicated that “they were satisfied with the training program”. Thus, this training program appears to be effective for improving student awareness and understanding of the prevention of primary osteoporosis. The results suggest that the students understood the importance of an educational intervention for local residents such as a simulated health class using their own bone density laboratory data.

## Conclusion

This experience-based education program that combined the measurement of bone density and preparation and presentation of a simulated health class improved the awareness and understanding of osteoporosis prevention in pharmacy students.

## Abbreviations

ADL: activities of daily living
BAR: bone area ratio
BMI: body-mass index
DXA: dual energy X-ray absorptiometry
PBM: peak bone mass
QOL: quality of life
QUS: quantitative ultrasound
SOS: speed of sound
